# Hierarchical Capability in Distinguishing Severities of Sepsis via Serum Lactate: A Network Meta-Analysis

**DOI:** 10.3390/biomedicines12020447

**Published:** 2024-02-17

**Authors:** Binlu Zhu, Ruixi Zhou, Jiangwei Qin, Yifei Li

**Affiliations:** Department of Pediatrics, West China Second University Hospital, Sichuan University, No. 20, 3rd Section, South Renmin Road, Chengdu 610041, China; binluzhu@163.com (B.Z.); zhouruixi0104@163.com (R.Z.); 2018141241084@stu.scu.edu.cn (J.Q.)

**Keywords:** lactate, sepsis, severe sepsis, septic shock, mortality, network meta-analysis

## Abstract

**Background:** Blood lactate is a potentially useful biomarker to predict the mortality and severity of sepsis. The purpose of this study is to systematically review the ability of lactate to predict hierarchical sepsis clinical outcomes and distinguish sepsis, severe sepsis and septic shock. **Methods**: We conducted an exhaustive search of the PubMed, Embase and Cochrane Library databases for studies published before 1 October 2022. Inclusion criteria mandated the presence of case–control, cohort studies and randomized controlled trials that established the association between before-treatment blood lactate levels and the mortality of individuals with sepsis, severe sepsis or septic shock. Data was analyzed using STATA Version 16.0. **Results:** A total of 127 studies, encompassing 107,445 patients, were ultimately incorporated into our analysis. Meta-analysis of blood lactate levels at varying thresholds revealed a statistically significant elevation in blood lactate levels predicting mortality (OR = 1.57, 95% CI 1.48–1.65, I^2^ = 92.8%, *p* < 0.00001). Blood lactate levels were significantly higher in non-survivors compared to survivors in sepsis patients (SMD = 0.77, 95% CI 0.74–0.79, I^2^ = 83.7%, *p* = 0.000). The prognostic utility of blood lactate in sepsis mortality was validated through hierarchical summary receiver operating characteristic curve (HSROC) analysis, yielding an area under the curve (AUC) of 0.72 (95% CI 0.68–0.76), accompanied by a summary sensitivity of 0.65 (95% CI 0.59–0.7) and a summary specificity of 0.7 (95% CI 0.64–0.75). Unfortunately, the network meta-analysis could not identify any significant differences in average blood lactate values’ assessments among sepsis, severe sepsis and septic shock patients. **Conclusions:** This meta-analysis demonstrated that high-level blood lactate was associated with a higher risk of sepsis mortality. Lactate has a relatively accurate predictive ability for the mortality risk of sepsis. However, the network analysis found that the levels of blood lactate were not effective in distinguishing between patients with sepsis, severe sepsis and septic shock.

## 1. Introduction

Sepsis, characterized by life-threatening organ dysfunction resulting from a dysregulated host response to infection [1], represents a significant global health concern, impacting millions of individuals worldwide. Several significant infectious conditions and inappropriate treatment may culminate in the progression to severe sepsis or septic shock [2]. To enhance clinical outcomes in these patients, early identification of individuals at risk of mortality and timely optimization of clinical decision-making are of paramount importance [3].

Clinical scoring systems, such as Sequential Organ Failure Assessment (SOFA) (Supplementary Table S1) and systemic inflammatory response syndrome (SIRS) (Supplementary Table S2), have been proposed for predicting sepsis-related outcomes, including mortality [4]. Nevertheless, SOFA entails a time-consuming process that necessitates multiple laboratory and clinical data inputs, while SIRS exhibits limited sensitivity in predicting mortality [5]. In contrast, blood lactate levels offer a rapid and easily obtainable measurement, serving as a surrogate for tissue hypoperfusion in critically ill patients [6]. Combining point-of-care lactate assessment with the qSOFA (Supplementary Table S3) score in rapid bedside assessments more accurately identifies the risk of sepsis-related mortality compared to using the qSOFA score alone [7].

**Figure 1:**
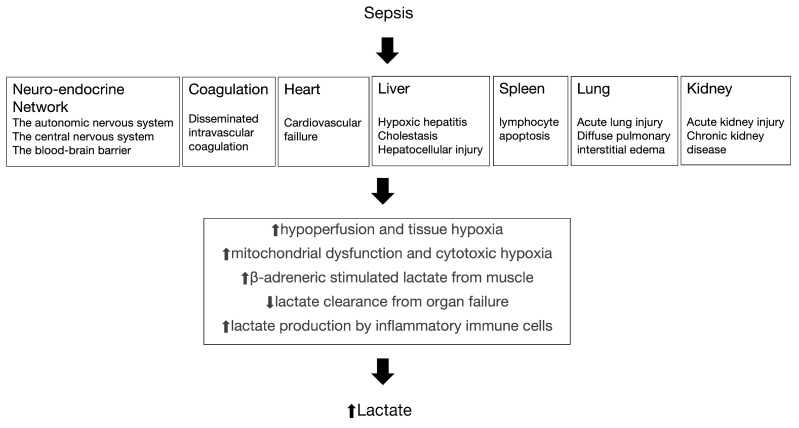
The pathogenesis of sepsis and the relationship with increased lactate.

Elevated blood lactate levels may result from tissue hypoxia and anaerobic metabolism; they can also develop in other ways (Figure 1) [8]. Studies consistently demonstrate that the duration and severity of hyperlactatemia are directly correlated with mortality in septic shock patients. Elevated blood lactate levels serve as a valuable marker for assessing the severity of sepsis and predicting patient outcomes [9,10,11]. This is attributed to their ability to accurately and promptly reflect the perfusion status of peripheral tissues in the body and their sensitivity in indicating the presence of cellular hypoxia or tissue hypoperfusion [12,13,14]. However, there exists inconsistency in the reference criteria for predicting the prognosis of sepsis in cases of elevated lactate levels. While certain studies emphasized an initial lactate level greater than 2.0 mmol/L as indicative of elevated lactate levels [15,16], others suggested that elevated lactate levels are defined by an initial lactate level exceeding 4.0 mmol/L [17,18,19]. Furthermore, there is no relevant quantitative meta-analysis focusing on different blood lactate level to predict different outcomes in sepsis, such as severe sepsis and septic shock.

For this purpose, we conducted a systematic review and diagnostic systematic review to rigorously and quantitatively assess the accuracy of blood lactate levels in predicting sepsis mortality. Additionally, we performed a network meta-analysis to evaluate whether blood lactate can differentiate between sepsis, severe sepsis and septic shock.

## 2. Methods

### 2.1. Study Protocol

This analysis was conducted in accordance with a predetermined protocol following the recommendations of a guideline for systematic reviews of prognostic factor studies. The Preferred Reporting Items for Systematic Reviews and Meta-analyses (PRISMA) Extension Statement for Reporting of Systematic Reviews Incorporating Network Meta-analyses of Health Care Interventions was followed for data abstractions. Our study protocol was registered on PROSPERO (CRD449572).

### 2.2. Search Strategy

We comprehensively searched the PubMed, Cochrane and Embase databases for English-language studies published before 1 October 2022. The strategy was “(“sepsis” [MeSH Terms] OR “sepsis” [All Fields] OR “septic shock” [MeSH Terms] OR “septic shock” [All Fields]) AND (“lactate” [MeSH Terms] OR “lactate” [All Fields] OR “hyperlactatemia” [All Fields])”.

### 2.3. Study Selection

Titles and abstracts of search results were screened independently (YL). The full texts of the remaining results were assessed independently by another two of us (BZ, RZ) for inclusion based on predetermined criteria. Any discrepancies were resolved through discussion, potentially with a third reviewer. We manually searched the reference lists of included studies and existing systematic reviews as well as all articles citing the included studies on Google Scholar.

In accordance with the objectives of our meta-analysis, we developed a ‘Population, Index prognostic factor, Comparator prognostic factor, Outcome, Timing, Settings’ (PICOTS) framework adapted from the guideline proposed by Riley et al. [20]. Our study inclusion criteria were as follows according to PICOTS framework: (1) population: sepsis patients with a well-defined diagnostic reference standard for sepsis; (2) index prognostic factor: before-treatment blood lactate levels measured; (3) outcome: nonsepsis, sepsis, severe sepsis, sepsis shock and death; (4) if studies were based on overlapping patients, the most completed one was chosen; and (5) studies were restricted to English publications. We used the following criteria for study exclusion: (1) studies lacking relevant outcomes or lactate levels; and (2) conference abstracts, reviews, case reports, and experiment studies.

### 2.4. Data Collection and Assessment of Study Quality

The relevant articles and eligible data were assessed and extracted by two authors (BZ, RZ), respectively. If a disagreement occurred, it was discussed and the consensus with a third author was reached. The quality of evidence was assessed by the modified Grading of Recommendations Assessment, Development, and Evaluation system (GRADE) by consensus among the authors [21,22].

The following data were collected from each study: first author name, area, publication date, the type of studied design, number of patients, timing of lactate measurements and primary outcome (nonsepsis, sepsis, severe sepsis, sepsis shock and death). When an included study reported different cut-off values, we chose one which made both sensitivity and specificity more than 50% as possible. When an included study reported the same outcome at different follow-up timepoint (e.g., 7-day mortality and 30-day mortality), we chose the earliest one. If the included studies did not report the mean and standard deviation, estimates for these parameters were derived from the sample size, median and quartiles [23,24]. Table 1 presents the baseline characteristics of included studies.

Two independent reviewers (BZ, RZ) performed quality assessments of selected studies using the QUADAS-2 criteria [25]. This checklist consists of four key domains: patient selection, index test, reference standard, and flow and timing. Within each study, the domains are assessed in terms of risk of bias and the first three of these domains are assessed in terms of concerns about applicability. Signaling questions as specified in the QUADAS-2 tool enable the reviewer to give each domain a rating of high, low or unclear. If the answers to all signaling questions for a domain are “yes”, then risk of bias can be judged low. If any signaling question is answered “no”, potential for bias exists. The “unclear” category should be used only when insufficient data are reported to permit a judgment. When there was a disagreement, the third author made the final decision based on the criteria. Details of the QUADAS-2 criteria are elaborated on Table 2.

Using the Newcastle–Ottawa Scale (NOS) [26] for cohort studies, the risk of bias was assessed for each outcome in all included studies. According to the selection of cohort (up to four points), the comparability of cohort design and analysis (up to two points) and the adequacy of result measurement (up to three points), a maximum of nine points will be obtained. Seven to nine points are considered high quality (low risk of bias) (Table 3).

### 2.5. Statistical Analysis

The meta-analysis used the combined effects of each result. We calculated odds ratios (ORs) and 95% confidence intervals (CIs) for each result using a random effects model, the between-study heterogeneity was evaluated by the χ^2^-based Q statistics and I^2^ test, and a significant heterogeneity was as *p* (value of Q test) < 0.1 or I^2^ > 50%. When significant heterogeneity was observed, we would apply the random effects models for analysis. Otherwise, we would apply the fixed effects models. We applied funnel plots as well as Egger’s test [27] to assess publication bias. A two-sided *p* value of 0.05 was deemed as statistical significance. Based on different classifications of blood lactate levels and the categorization of children and adults, we conducted subgroup analyses.

We performed meta-analysis by using the hierarchical summary receiver operating characteristic (HSROC) model to estimate and compare SROC curves [28]. Sensitivity and specificity were calculated by true positives, false positives, true negatives, and false negatives. In order to further quantify the lactate level of various outcome, we calculated the frequentist analogue of the surface under the cumulative ranking curve (SUCRA) for each outcome [29].

Data was analyzed using STATA Version 16.0 [30]. The network was evaluated using frequentist multivariate meta-analysis (commands network meta and mvmeta) in Stata 16.0. Additionally, publication bias and sensitivity analysis were also conducted by STATA version 16.0.

## 3. Results

**Figure 2:**
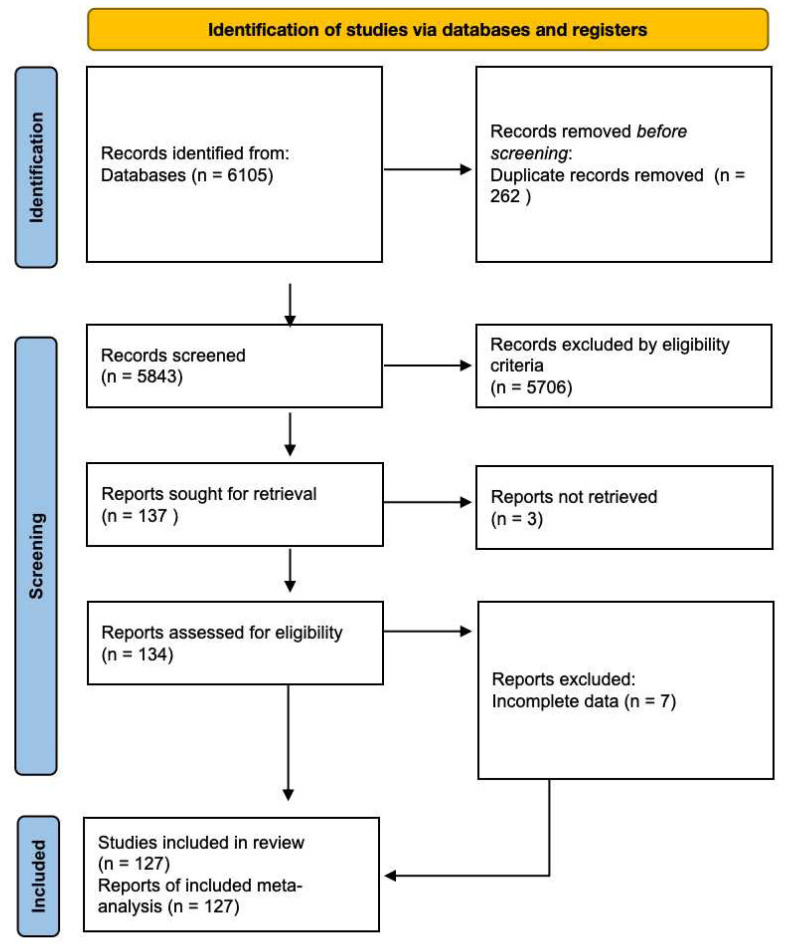
Preferred Reporting Items for Systematic Reviews and Meta-Analyses (PRISMA) flow diagram for study identification and selection.

A total of 6105 articles were initially retrieved from the databases. However, after a rigorous selection process, 127 studies involving a cumulative cohort of 107,445 patients were ultimately incorporated into our analysis (Figure 2). No additional pertinent articles were found in the reference lists of the original publications. Detailed characteristics of the included studies can be found in Table 1. Among the included studies, before-treatment blood lactate measured values had been documented in 78 studies [6,9,10,11,12,13,15,16,17,18,19,31,32,33,34,35,36,37,38,39,40,41,42,43,44,45,46,47,48,49,50,51,52,53,54,55,56,57,58,59,60,61,62,63,64,65,66,67,68,69,70,71,72,73,74,75,76,77,78,79,80,81,82,83,84,85,86,87,88,89,90,91,92,93,94,95,96,97]. Also, most of them provided data on the association between nonsepsis, sepsis, severe sepsis and septic shock and different blood lactate values. Meanwhile, there were 82 studies that demonstrated the relationship between blood lactate and sepsis induced mortality [9,11,15,19,31,34,36,38,39,40,42,43,44,45,46,47,48,49,50,51,53,55,58,59,62,63,65,67,68,69,70,71,72,74,75,76,78,81,83,85,87,88,89,91,93,95,97,98,99,100,101,102,103,104,105,106,107,108,109,110,111,112,113,114,115,116,117,118,119,120,121,122,123,124,125,126,127,128,129,130,131,132]. Additionally, there were 46 articles reported the diagnostic accuracy of blood lactate levels in determining sepsis and associated clinical prognosis [11,15,31,33,34,38,43,44,45,50,53,56,59,64,65,66,67,69,70,71,72,77,78,85,89,100,102,103,106,107,108,110,111,114,117,121,122,124,133,134,135,136,137,138,139,140]. All included studies were observational studies.

In the pooled analysis, the recommended threshold for identifying the high level of blood lactate varied across the included studies. Nine studies set a cut-off value for high level blood lactate below 2 mmol/L [33,38,41,85,103,108,124,135,140], while 36 studies recognized the cut-off values for high level blood lactate between 2 and 4 mmol/L [6,11,15,16,34,35,39,43,50,52,53,64,65,66,67,69,70,71,72,76,77,89,94,100,102,106,107,110,111,114,117,121,133,134,138,139]. However, there were 19 studies that used a significant elevated cut-off values in demonstrating high blood lactate levels above 4 mmol/L [6,13,17,18,19,42,43,56,57,63,65,69,78,80,93,94,95,96,97].

### 3.1. Assessment of Methodological Quality

The QUADAS-2 tool was employed to evaluate the quality of the included studies. The majority of these studies met a significant portion of the criteria outlined in the QUADAS list. Detailed results of the QUADAS assessments are provided in Table 2. Among the enrolled studies, a total of 124 studies were included, with 110 of them achieving a NOS score equal to or greater than seven points, indicating their classification as high-quality studies. Please refer to Table 3 for a comprehensive overview of these high-quality studies.

### 3.2. Higher Blood Lactate Value Was Associated with Mortality of Sepsis

**Figure 3:**
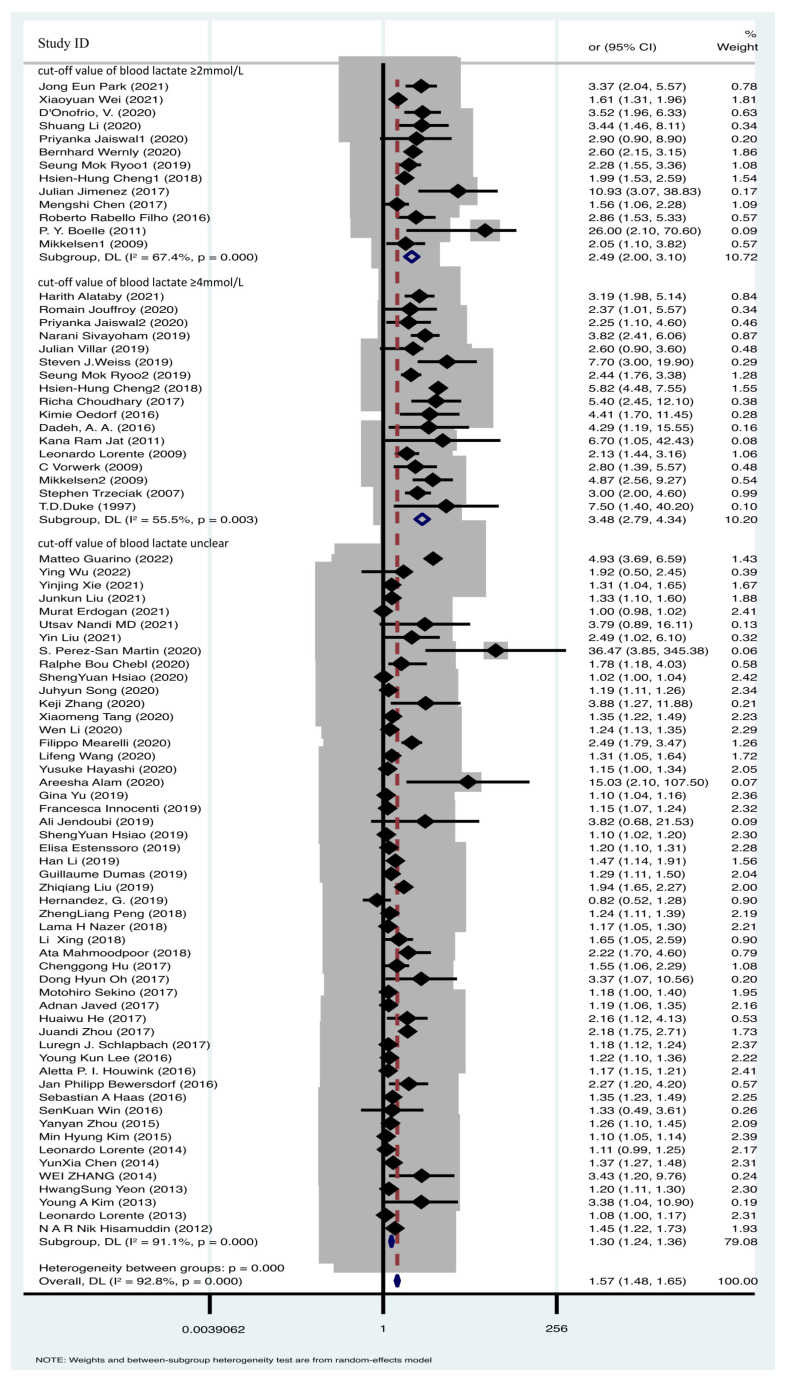
Forest plot of lactate and sepsis mortality [6,9,10,11,12,13,15,16,17,18,19,31,32,33,34,35,36,37,38,39,40,41,42,43,44,45,46,47,48,49,50,51,52,53,54,55,56,57,58,59,60,61,62,63,64,65,66,67,68,69,70,71,72,73,74,75,76,77,78,79,80,81,82,83,84,85,86,87,88,89,90,91,92,93,94,95,96,97].

A total of 78 articles were included in the comparative analysis, assessing the impact of various blood lactate levels on sepsis prognosis. Heterogeneity testing revealed significant heterogeneity across the research findings (I^2^ = 92.8%, *p* = 0.000). To account for this heterogeneity, a random effects model was applied for the meta-analysis, confirming the prognostic significance of elevated blood lactate levels in sepsis mortality [OR = 1.57, 95% CI 1.48–1.65] (Figure 3). This finding indicates that higher blood lactate levels are associated with sepsis mortality. Given the substantial heterogeneity, we conducted a subgroup analysis based on different blood lactate cutoff values. Specifically, 13 studies utilized a blood lactate cutoff of ≥2 mmol/L (OR = 2.49, 95% CI 2.00–3.10, I^2^ = 67.4%, *p* = 0.000), while 17 studies employed a cutoff of ≥4 mmol/L (OR = 3.48, 95% CI 2.79–4.34, I^2^ = 55.5%, *p* = 0.003) (Figure 3). Despite the presence of considerable heterogeneity, the pooled effect sizes remained robust, as confirmed by sensitivity analysis (Supplementary Figure S1). Such data demonstrated the blood lactate value was positive associated with sepsis mortality.

### 3.3. Blood Lactate Significantly Elevated in Non-Survivors of Sepsis Events

**Figure 4:**
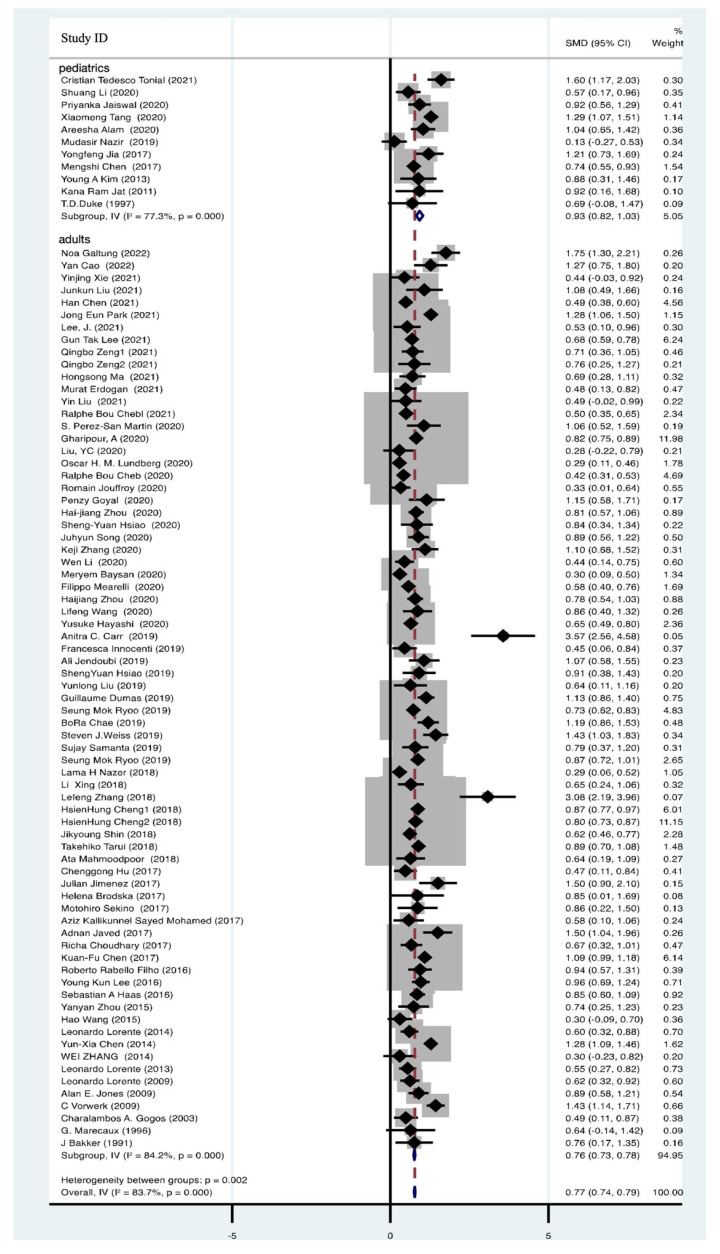
Forest plot of lactate in non-survivors of sepsis [9,11,15,19,31,34,36,38,39,40,42,43,44,45,46,47,48,49,50,51,53,55,58,59,62,63,65,67,68,69,70,71,72,74,75,76,78,81,83,85,87,88,89,91,93,95,97,98,99,100,101,102,103,104,105,106,107,108,109,110,111,112,113,114,115,116,117,118,119,120,121,122,123,124,125,126,127,128,129,130,131,132].

A total of 82 articles, comprising 46,956 participants, provided data on blood lactate levels among both survivors and non-survivors with sepsis. These data unequivocally demonstrated a significant elevation in blood lactate levels among non-survivors (SMD = 0.77, 95% CI 0.74–0.79, I^2^ = 83.7%, *p* = 0.000) (Figure 4). To gain further insights, we conducted subgroup analyses, with 11 studies focusing on pediatric patients (SMD = 0.93, 95% CI 0.82–1.03, I^2^ = 77.3%, *p* = 0.000) and 71 studies on adult patients (SMD = 0.76, 95% CI 0.73–0.78, I^2^ = 84.2%, *p* = 0.000) (Figure 4). Despite substantial heterogeneity, the pooled effect sizes remained robust, as confirmed by sensitivity analysis (Supplementary Figure S2). Examination of funnel plots indicated no evidence of publication bias, as they exhibited a symmetrical distribution (Supplementary Figure S3).

### 3.4. High Level Blood Lactate Demonstrated a Sufficient Prognostic Value in Determining the Sepsis Mortality

**Figure 5:**
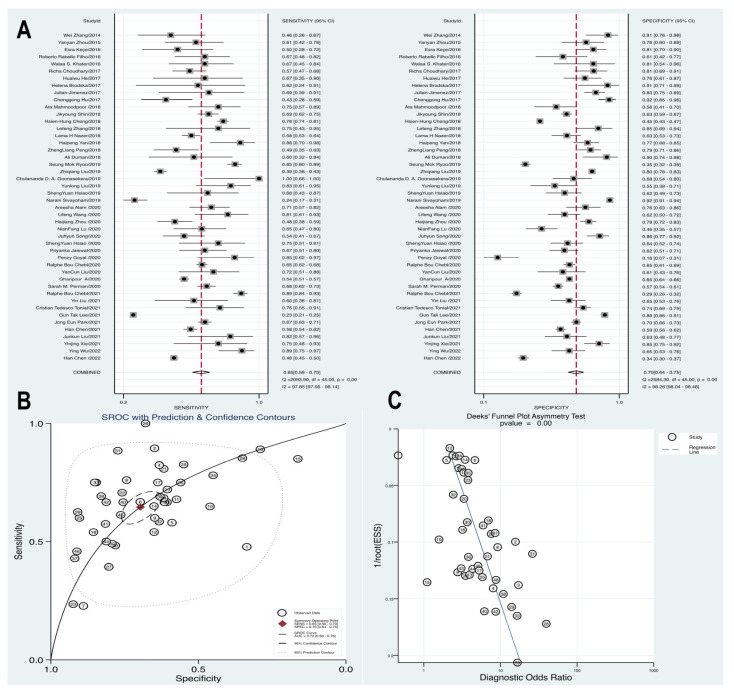
Forest plot of pooled sensitivity, specificity (**A**) and HSROC (**B**) for blood lactate levels predicting mortality **in** patients with sepsis. (**C**) Funnel plot with Deek’s test for diagnostic analysis between blood lactate levels and mortality [11,15,31,33,34,38,43,44,45,50,53,56,59,64,65,66,67,69,70,71,72,77,78,85,89,100,102,103,106,107,108,110,111,114,117,121,122,124,133,134,135,136,137,138,139,140].

We included 46 articles that focused on prognostic analysis, enabling the conversion of data from these studies into a fourfold table of diagnostic tests to assess the prognostic value of blood lactate in high-risk sepsis populations. A diagnostic meta-analysis was conducted to further investigate the prognostic role of blood lactate. The summary sensitivity was calculated at 0.65 (95% CI 0.59–0.70), with substantial heterogeneity observed (*p* = 0.00, Q = 2093.9, I^2^ = 97.85%). Similarly, the summary specificity was 0.7 (95% CI 0.64–0.75), and the pooled estimation indicated significant heterogeneity (*p* = 0.00, Q = 2584.3, I^2^ = 98.26%). The Hierarchical Summary Receiver Operating Characteristic (HSROC) curve demonstrated the potential prognostic value of blood lactate levels for high-risk sepsis patients, with an AUC of 0.72 (95% CI 0.68–0.76). Furthermore, the presence of asymmetric distribution in funnel plots, as indicated by Deek’s test (*p* = 0.00), raised the possibility of publication bias within the studies (Figure 5).

### 3.5. Blood Lactate Levels Failed to Distinguish Sepsis, Severe Sepsis and Septic Shock Based on Network Meta-Analysis

**Figure 6:**
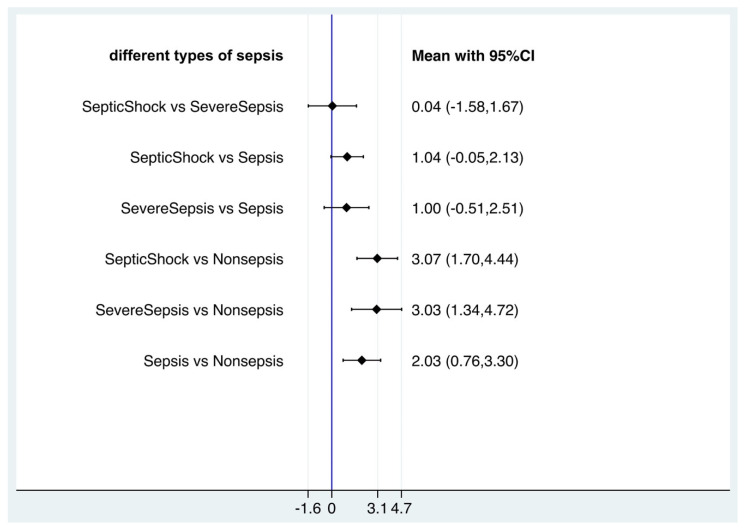
Forest plot of network meta-analysis of lactate and sepsis, severe sepsis and septic shock.

**Figure 7:**
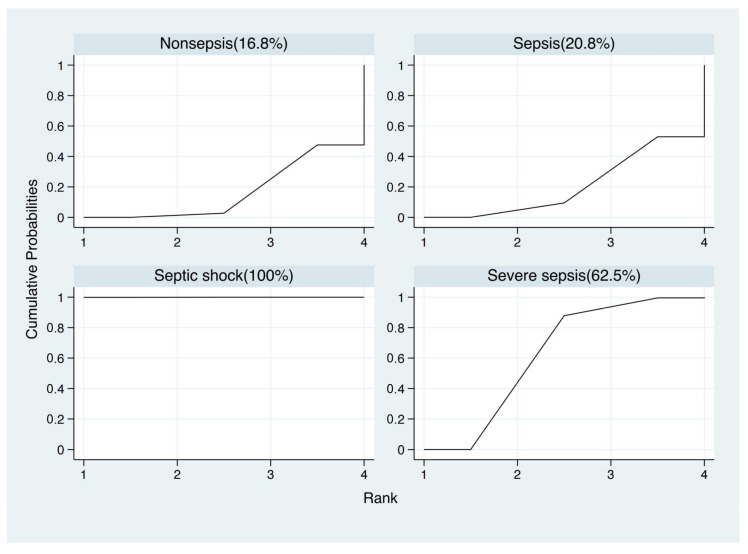
Results of network rank test and the surface under the cumulative ranking curve (SUCRA).

As the blood lactate level could indicate the prognosis of sepsis, we attempt to underline whether the value of blood lactate was correlated different outcomes of sepsis (sepsis, severe sepsis and septic shock). For that, network meta-analysis was used to compare the average blood lactate values among non-septic patients, septic patients, severe septic patients and septic shock patients with frequentist statistics (Supplementary Figure S4). In the network meta-analysis, there were significant differences between nonsepsis and sepsis, severe sepsis, septic shock (sepsis vs. nonsepsis, mean 2.03, 95% CI 0.76, 3.30, *p* < 0.005; severe sepsis vs. nonsepsis, mean 3.03, 95% CI 1.34, 4.72, *p* < 0.001; septic shock vs. nonsepsis, mean 3.07, 95% CI 1.70, 4.44, *p* < 0.001), but there were no significant differences among sepsis, severe sepsis and septic shock (severe sepsis vs. sepsis, mean 1.00, 95% CI −0.51, 2.51; septic shock vs. sepsis, mean 1.04, 95% CI −0.05, 2.13; septic shock vs. severe sepsis, mean 0.04, 95% CI −1.58, 1.67) (Figure 6). However, in SUCRA statistical analysis, it still showed the ranked blood lactate levels among patients with sepsis, severe sepsis and septic shock, with blood lactate levels in septic shock patients ranked first, followed by severe sepsis patients and sepsis patients (Figure 7). Funnel plots suggested no publication bias based on its symmetry (Supplementary Figure S4).

## 4. Discussion

Our study conclusively establishes an association between elevated blood lactate levels and increased mortality, providing updated statistical conclusions and risk values. Importantly, this association holds true across diverse demographic factors such as age, gender, race, geographic region, and the specific assay method employed to measure blood lactate. Furthermore, diagnostic systematic review results demonstrate the significant predictive ability of lactate in sepsis mortality. Higher cutoff values for lactate are associated with increased mortality risk. However, this network analysis did not underline any significant differences in average blood lactate levels among individuals with sepsis, severe sepsis and septic shock. In clinical practice, we cannot overly rely on lactate to determine the severity of sepsis. Mildly elevated lactate levels may also progress to severe sepsis or septic shock, and excessively high lactate levels may indicate non-septic infections. These complex clinical scenarios require a more flexible and nuanced approach to assessment.

Several recent studies have highlighted the potential prognostic markers of outcome in severe sepsis, including the central venous minus arterial carbon dioxide pressure to arterial minus central venous oxygen content ratio (Pcv-aCO2/Ca-cvO2 [141,142], the CRP/albumin ratio [86,143], and lactate clearance [65,113,116,144]. The diagnostic performance of the Pcv-aCO2/Ca-cvO2 ratio > 1.696 at 24 h was analyzed using the ROC curve, and it was found to have an AUC of 0.82 (95% CI, 0.661–0.979). The lactate > 1.6 mmol/L had an AUC of 0.853 (95% CI 0.712–0.915) for predicting 28-day mortality [141]. The CRP/albumin ratio had an AUC of 0.621 for predicting mortality [86]. Other biomarkers, such as first urine liver-type fatty acid binding protein (L-FABP), plasma mtDNA also had a predictive value for sepsis mortality [138,145]. The AUC of urine L-FABP and plasma mtDNA for mortality were 0.647 and 0.726, respectively. The AUC was 0.864 for lactate [138]. Lactate had the greatest association with mortality. However, these markers necessitate multiple laboratory measurements, which can be unfavorable for early and accurate assessment. Blood lactate levels are considered sensitive markers for sepsis and septic shock, reflecting cellular metabolism [146]. In our analysis, we included a larger number of studies, enhancing the comprehensiveness and reliability of our results.

In our analysis, we observed heterogeneity among the included studies. The levels of blood lactate varied significantly across different studies, resulting in substantial unmanageable heterogeneity in the pooled effects. This heterogeneity can be attributed, in part, to the diverse sources of arterial and venous blood lactate, variations in measurement equipment and assays, as well as differences in methodologies for lactate measurements and the use of various lactate cut-off values. Moreover, even after conducting subgroup analysis, high heterogeneity persisted due to significant disparities in diagnostic criteria, primary diseases, disease severity, and treatment status, necessitating further validation and analysis of clinical trial results. It is important to note that blood lactate levels in the body are influenced by multiple processes, including lactate generation, transformation, clearance, and recycling [8]. The dynamics of blood lactate levels can provide valuable insights into identifying individuals at high risk for poor clinical outcomes. However, our study has several limitations. It underscores that a single measurement of blood lactate levels in clinical practice may not fully capture the dynamic state of the body. Therefore, dynamic monitoring of blood lactate levels is recommended for more effectively guiding sepsis treatment and assessing prognosis. Additionally, a model incorporating more variables may offer improved predictive capability compared to a single lactate variable. Recently, a study suggested that compared to lactate and albumin alone, the predictor value of the lactate and albumin ratio was outstanding in predicting death and hospital stay (discharge) among sepsis participants, with a sensitivity of 100% and a specificity of 88% [147]. As most of the included studies are observational, they cannot infer causation. Further research, ideally through controlled trials or experimental studies, is needed to establish causal relationships between blood lactate levels and sepsis outcomes. While lactate plays a significant role in predicting the risk of sepsis mortality, in clinical practice, it is essential to consider other clinical indicators and the overall condition of the patient. This comprehensive approach allows for a more thorough evaluation of the patient’s condition and the development of appropriate treatment plans.

## 5. Conclusions

Based on the meta-analysis, blood lactate revealed the capability to predict the multiple sepsis mortality. This study demonstrated the high-level blood lactate was associated with an elevated risk of death and predicted higher risk of mortality. However, the levels of blood lactate failed to distinguish sepsis, severe sepsis and septic shock. Large-scale multicenter randomized clinical trials are needed to provide high-level evidence and confirm the optimal cut-off for prognosis of sepsis. In clinical practice, we cannot overly rely on lactate to determine the severity of sepsis; the dynamic monitoring of blood lactate levels is recommended for more effectively guiding sepsis treatment and assessing prognosis.

## Figures and Tables

**Table 1:** Basic characteristics of included studies.

Author	Area	Year	Sepsis Criterion	Outcome	Study Design	Number of Patients	Number of Sepsis	Number of Deaths	Timing of Measurements	Comparisons	Assessment *
Han Chen	China	2022	Sepsis-3	28-day mortality	MIMIC-IV database	21,333	4219	17,114	within 24 h of ICU admission	Sepsis vs. Non-sepsis	a, d
Lincui Zhong	China	2022	Sepsis-3	In-ICU mortality	retrospective	311	203	108	within 2 h of ICU admission	Sepsis vs. septic shock	a
Noa Galtung	Germany	2022	Sepsis-3	in-hospital mortality	prospective	301	279	22	admission to ED	survival vs. death	a, b
Matteo Guarino	Italy	2022	diagnosis	in-hospital mortality	retrospective	1001	556	218	admission to ED		c
Yan Cao	China	2022	Sepsis-3	28-day mortality	prospective	86	65	21	within 24 h of ED admission	survival vs. death	a, b
Ying Wu	China	2022	2013 SSC	28-day mortality	Case-control	112				Sepsis vs. severe sepsis vs. septic shock vs. controls	a, c, d
Yinjing Xie	China	2021	Sepsis-3	28-day mortality	retrospective	90	67	23	admission to ED	survival vs. death	a, b, c, d
Harith Alataby	USA	2021	ICD-10 code	30-day mortality	retrospective	427		149	within 24 h of hospital admission		c
Junkun Liu	China	2021	Sepsis-3	28-day mortality	prospective	66	49	17	within 24 h of ICU admission	survival vs. death	a, b, c, d
Han Chen	China	2021	Sepsis-3	28-day mortality	MIMIC-III	1371	826	545	within 24 h of ICU admission	survival vs. death	b, d
Jong Eun Park	Korea	2021	Sepsis-3	28-day mortality	prospective	755	635	102	in ED or hematology-oncology department or ICU	survival vs. death	a, b, c, d
Jongmin Lee	Korea	2021	Diagnosis	in-hospital mortality	prospective	88	50	38	Day 1 of hospital admission	survival vs. death	b
Gun Tak Lee	Korea	2021	Diagnosis	28-day mortality	retrospective	2568	1977	591	admission to ED	survival vs. death	a, b, d
Cristian Tedesco Tonial	Brazil	2021	Diagnosis and SIRS	in-hospital mortality	retrospective	294	267	25	Highest within 24 h of PICU admission	survival vs. death	b, d
Xiaoyuan Wei	China	2021	Diagnosis	30-day mortality	retrospective	2948		956	admission to to ICU		c
Xiaonan Chen	China	2021	ICD-9 code	in-hospital mortality	MIMIC-III	4555	1712	2843	first of ICU admission	survival vs. death	a
Qingbo Zeng	China	2021	Sepsis-3	90-day mortality	retrospective	Training 161 Validation 70	112 46	49 24	within 24 h of ICU admission	survival vs. death	b
Hongsong Ma	China	2021	Diagnosis	in-hospital mortality	retrospective	127		31		Mild vs. severe vs. Sepsis shock survival vs. death	a, b
Murat Erdogan	Turkey	2021	Sepsis-3	28-day mortality	prospective	148	96	52		survival vs. death	a, b, c
Utsav Nandi	USA	2021	SIRS	in-hospital mortality	retrospective	160	127	33	admission to ED	survival vs. death	a, c
Yin Liu	China	2021	2016 SSC	28-day mortality	retrospective	91	71	20	Day 1 of ICU admission	survival vs. death	b, c, d
Ralphe Bou Chebl	Lebanon	2021	Sepsis-3	in-hospital mortality	prospective	939	720	219	admission to ED	survival vs. death	a, b, d
Valentino D’Onofrio	Netherlands	2020		in-hospital mortality	prospective	1690	90	1600	admission to ED	survival vs. death	c
Shuang Li	China	2020	Diagnosis	90-day mortality	retrospective	146	113	33	within 24 h after the collection of blood culture samples	Survivors vs. death	a, b, c
S. Perez-San Martin	Spain	2020	Sepsis-3	in-hospital mortality	prospective	75	54	21	admission to ICU	Survivors vs. death	a, b, c
Sarah M. Perman	U.S.A	2020	Sepsis-3	in-hospital mortality	retrospective	2859			admission to ED		d
Amin Gharipour	Australia	2020	MIMIC-III	28-day mortality	retrospective	6414	5364	1050	first 24 h of ICU admission	Survivors vs. death	a, b, d
Yancun Liu	China	2020	Sepsis-3	28-day mortality	prospective	63	38	25	within 12 h after EICU admission	Survivors vs. death	a, b, d
Oscar H. M. Lundberg	Sweden	2020	Sepsis-3	30-day mortality	retrospective	632	458	174	admission to ICU	Survivors vs. death	b
Ralphe Bou Chebl ^1^	Lebanon	2020	Sepsis-3	in-hospital mortality	retrospective	1381	575	806	admission to ED	Survivors vs. death	a, b, d
Ralphe Bou Chebl ^2^	Lebanon	2020	Sepsis-3	in-hospital mortality	retrospective	1627			admission to ED	lactate levels	a, c
Romain Jouffroy	France	2020	SFAR-SRLF *	30-day mortality	prospective	177	118	59	first ICU admission	Survivors vs. death	a, b, c
Penzy Goyal	India	2020	Sepsis-3	In-ICU mortality		63	43	20	admission to ICU	Survivors vs. death	a, b, d
Haijiang Zhou	China	2020	Sepsis-3	28-day mortality	retrospective	340	250	90	admission to ED	Survivors vs. death	a, b
Tae Sik Hwang	Korea	2020		in-hospital mortality	retrospective	165			admission to ED	septic shock	a
Priyanka Jaiswal	India	2020	SIRS	in-hospital mortality	prospective	149	104	45	admission to ED	Survivors vs. death	b, c, d
ShengYuan Hsiao	Taiwan	2020	Sepsis-3	in-hospital mortality	prospective	100	80	20	within 24 h of ED admission	Survivors vs. death	b, c, d
Juhyun Song	Korea	2020	Sepsis-3	28-day mortality	prospective	160	97	63	within 6 h of the clinical diagnosis of sepsis	Survivors vs. death	b, c, d
Keji Zhang	China	2020	Sepsis-3	in-hospital mortality	retrospective	185	158	27	first EICU admission	Survivors vs. death	b, c
Nianfang Lu	China	2020	Sepsis-3	in-hospital mortality	prospective	126	89	37	within 24 h of ICU admission	Survivors vs. death	d
Xiaomeng Tang	China	2020	Diagnosis	in-hospital mortality	Database	819	720	99	first batch of data after PICU admission	Survivors vs. death	a, b, c
Wen Li	China	2020	Sepsis-3	in-hospital mortality	prospective	626	378	248	admission to ICU	Survivors vs. death	a, b, c
Meryem Baysan	Netherlands	2020	APACHE IV	in-hospital mortality	retrospective	451	291	160	first ICU admission	Survivors vs. death	a, b
Filippo Mearelli	Italy	2020	Sepsis-3	30-day mortality	prospective	828	148	680	admission to ED	Survivors vs. death	b, c
Haijiang Zhou	China	2020	Sepsis-3	28-day mortality	retrospective	336	247	89	admission to ED	Survivors vs. death	a, b, d
Lifeng Wang	China	2020	Sepsis-3	28-day mortality		107	81	26	admission to ED	Survivors vs. death	a, b, c, d
Yusuke Hayashi	Japan	2020		in-hospital mortality	MIMIC-III	781	523	258	admission to ICU	Survivors vs. death	a, b, c
Bernhard Wernly	Austria	2020	Diagnosis	ICU mortality	MIMIC-III	5586	3293	2293	first 24 h of ICU admission	no acidosis vs. acidosis	c
Areesha Alam	India	2020	International pediatric SCC	Early Mortality ≤48h	prospective	116	58	58	within 30 min of admission	Survivors vs. death	b, c, d
Gina Yu	Korea	2019	Diagnosis	28-day mortality	retrospective	362	247	115	within 12 h of ED admission	Survivors vs. death	c
Anitra C. Carr	New Zealand	2019		in-hospital mortality		44	32	12	admission to ICU	Survivors vs. death	a, b
Mudasir Nazir	India	2019	International pediatric SCC	60-day mortality	prospective	112	77	35	admission to PICU	Survivors vs. death	a, b
Francesca Innocenti	Italy	2019	2001SCCM/ESICM/ACCP/ATS/SIS	in-hospital mortality	prospective	268	153 74	115 41	admission to ED	without shock vs. Sepsis shock Survivors vs. death	a, b, c
Narani Sivayoham	UK	2019	Red Flag/SIRS	in-hospital mortality	prospective	1078	938	140	admission to ED/ICU	Survivors vs. death	c, d
Julian Villar	USA	2019	ICD-9	30-day mortality	retrospective	3325	546	2779	admission to ED/ICU	Sepsis vs. non-sepsis	a, c
Ali Jendoubi	Tunisia	2019	ACCP/SCCM	28-day mortality	prospective	75	34	41	admission to ICU	Survivors vs. death	a, b, c
Jie Jiang	China	2019	Sepsis-3	in-hospital mortality	retrospective	100	77	23	within 24 h of ICU admission	Sepsis vs. non-sepsis	a
Shengyuan Hsiao	Taiwan	2019	Sepsis-3	in-hospital mortality	prospective	126	39 16 68	87 71 19	first 24 h of ED admission	Control vs. Sepsis Sepsis vs. Sepsis shock Survivors vs. death	a, b, c, d
Elisa Estenssoro	Argentina	2019	Sepsis-3	in-hospital mortality	prospective	367 443			within 24 h of ICU admission	Public hospitals vs. Private hospitals	a, c
Yunlong Liu	China	2019	Sepsis-3	28-day mortality	prospective	63	40	23	Within 24 h after diagnosis	Survivors vs. death	b, d
Anibal Basile-Filho	Brasil	2019	Sepsis-3	in-hospital mortality	retrospective	83	35	48	first 24 h after ICU admission	Survivors vs. death	a
Han Li	China	2019	diagnosis	in-hospital mortality		245	183	62	admission to hospitals	Survivors vs. death	c
Guillaume Dumas	France	2019	2001SCCM/ESICM/ACCP/ATS/SIS	14-day mortality	prospective	256	164	95	at 0, 12, 24 h after ICU admission	Survivors vs. death	b, c
Seung Mok Ryoo1	Korea	2019	Goal-directed resuscitation	28-day mortality	prospective	2102	1653	449	within 24 h after ED admission	Survivors vs. death	a, b
BoRa Chae	Korea	2019	SIRS	30-day mortality	retrospective	301	258	43	admission to ED	Survivors vs. death	a, b
Chulananda D. A. Goonasekera	Turkey	2019	international consensus conference	28-day mortality	retrospective	62	53	9	admission to PICU	Survivors vs. death	d
Steven J.Weiss	USA	2019	diagnosis	in-hospital mortality	retrospective	351	323	28	admission to ICU	Survivors vs. death	b, c
Sujay Samanta	India	2019		28-day mortality	prospective	104	36	68	within 24 h of ICU admission	Survivors vs. death	b
Zhiqiang Liu	China	2019	diagnosis	30-day mortality 90-day mortality hospital mortality 1-year mortality	MIMIC III	1865	1166	699	first 24 h from ICU admission	Lactate < 3.225 vs. Lactate ≥ 3.225	a, c, d
Glenn Hernández	Chile	2019	Sepsis-3	28-day mortality	randomized clinical trial	212	115	97	admission to the ICU	Survivors vs. death	a, c
Seung Mok Ryoo2	Korea	2019	Sepsis-3	28-day mortality	retrospective	1060	795	265	initial and 6 h from septic shock recognition	Survivors vs. death	b, c, d
Ali Duman	Aydin	2018	Sepsis-3	30-day mortality	prospective	46	46		admission to ED	Infection vs. sepsis	a, d
Zhengliang Peng	China	2018	Sepsis-3	30-day mortality	retrospective	166	11	55	admission to EICU	Survivors vs. death	a, c, d
Haipeng Yan	China	2018	2012SSC	in-hospital mortality	case–control	183	70 30	53 30	1 h of the hospital admission	Sepsis vs. severe sepsis vs. non-sepsis vs. health	a, d
Lama H Nazer	Jordan	2018	Sepsis-2	in-hospital mortality	retrospective	401	98	303	admission to ICU	Survivors vs. death	a, b, c, d
Li Xing	China	2018	Sepsis-3	28-day mortality	prospective	120	88	32	within 24 h after diagnosis	Survivors vs. death	b, c
Lefeng Zhang	China	2018	SCCM/ESICM	28-day mortality	retrospective	51	39	12	admission to ICU	Survivors vs. death	b, d
HsienHung Cheng	China	2018	ICD-9	28-day mortality	retrospective	7087	5414	1673	within 6 h of ED admission	Survivors vs. death	b, c, d
Jikyoung Shin	Korea	2018	diagnosis	28-day mortality	retrospective	946	733	213	admission to ED	Survivors vs. death	a, b, d
Takehiko Tarui	Japan	2018	2001SCCM/ESICM/ACCP/ATS/SIS	in-hospital mortality	prospective	554	399	155	Worst lactate during the initial 24 h	Survivors vs. death	b
Ata Mahmoodpoor	Iran, USA	2018	diagnosis	28-day mortality	prospective	82	50	32	within 24 h of ICU admission	Survivors vs. death	b, c, d
Chenggong Hu	China	2017	Sepsis-3	28-day mortality		141	99	42	day 0, 3, 7 of hospitalization	Survivors vs. death	b, c, d
Julian Jimenez	Spain	2017	Sepsis-3	30-day mortality	prospective	136	123	13	initial admission to ED	Survivors vs. death	b, c, d
Helena Brodska	USA	2017	1992ACCP/SCCM	28-day mortality		30	22	8	day 1, 2, 3 of ICU admission	Survivors vs. death	a, b, d
Yongfeng Jia	China	2017	diagnosis	in-hospital mortality	retrospective	90	61	29	admission to PICU	Survivors vs. death	b
Dong Hyun Oh	South Korea	2017	2012SSC	28-day mortality	retrospective	1022	653	369	admission to ED	High lactate vs. Low lactate	c
Motohiro Sekino	Japan	2017	2001SCCM/ESICM/ACCP/ATS/SIS	28-day mortality	prospective	57	44	13	within 24 h of ICU admission	Survivors vs. death	a, b, c
Aziz Kallikunnel Sayed Mohamed	India	2017	SIRS	in-hospital mortality	prospective	80	26	54	admission to ICU	Survivors vs. death	b
Adnan Javed	USA	2017	diagnosis	24 h mortality	prospective	410	390	20	admission to ED	Survivors vs. death	a, b, c
Mengshi Chen	China	2017	Sepsis-3	in-hospital mortality	retrospective	592	438	154	within 24h of PICU admission	Survivors vs. death	b, c
Huaiwu He	China	2017	2001SCCM/ESICM/ACCP/ATS/SIS	ICU mortality	clinical investigation	61	49	12	admission to ICU	Survivors vs. death	a, c, d
Richa Choudhary	India	2017	2005pediatric SCC	in-hospital mortality	prospective	148	54	94	0 h, 24 h, 48 h of PICU admission	Survivors vs. death	b, c, d
Juandi Zhou	China	2017	2001SCCM/ESICM/ACCP/ATS/SIS	28-day mortality	retrospective	144			after the first 6 h of resuscitation		c
Luregn J. Schlapbach	Australia	2017	2005pediatric SCC	30-day mortality	multicenter binational cohort study	1697			admission to ICU		c
KuanFu Chen	Taiwan	2017	ICD-9	in-hospital mortality	retrospective	7011	6532	479	admission to ED	Survivors vs. death	a, b
Kimie Oedorf	Israel	2016	diagnosis	in-hospital mortality	prospective	488	202	286	during their ED stay	Without infection vs. With infection	c
Walaa S. Khater	Egypt	2016	2001SCCM/ESICM/ACCP/ATS/SIS	in-hospital mortality	prospective	80	40	40	admission to ICU	Sepsis vs. control	d
Roberto Rabello Filho	Brazil	2016	SSC	30-day mortality	retrospective	260	227	33	first 24 h of ED admission	Survivors vs. death	b, c, d
Ar-aishah Dadeh	Thailand	2016	SIRS	28-day mortality	prospective	131	34	97	within 24 h and at day 3 of ED admission	Septic shock vs. non-septic shock	a, c
Esra Keçe	Turkey	2016	SIRS	28-day mortality	prospective case–control	86	64	22	admission to ED	Sepsis vs. non-sepsis	d
Young Kun Lee	Korea	2016	2001SCCM/ESICM/ACCP/ATS/SIS	28-day mortality	retrospective	363	298	65	admission to ED	Survivors vs. death	a, b, c
Aletta P. I. Houwink	Netherlands	2016	diagnosis	ICU mortality	retrospective	821			first 24 h after admission		c
Jan Philipp Bewersdorf	Netherlands	2016	1992ACCP/SCCM	28-day mortality	prospective	440			admission to ED		a, c
Sebastian A Haas	Germany	2016		ICU mortality	retrospective	400	87	313		Survivors vs. death	b, c
Sen Kuan, Win	Singapore	2016		in-hospital mortality	open label randomized controlled trial	122	61	61		Intervention vs. control	c
Yanyan Zhou	China	2015	2001SCCM/ESICM/ACCP/ATS/SIS	in-hospital mortality		69	38	31	within the first 24 h of ICU admission	Survivors vs. death	b, c, d
Min Hyung Kim	Korea	2015	2004SSC	180-day mortality	retrospective	690			first 24 h of ED admission		c
Ivo Casagranda	Italy	2015	2001SCCM/ESICM/ACCP/ATS/SIS	7-day mortality 30-day mortality	prospective	130	59	71	admission to ED	Sepsis vs. severe sepsis or septic shock	a
Hao Wang	China	2015	2008SSC	28-day mortality	prospective	115	38	77	within the first 30 min after ICU admission	Survivors vs. death	b
Leonardo Lorente	Spain	2014	2001SCCM/ESICM/ACCP/ATS/SIS	30-day mortality	prospective	224	144	80	at the time severe sepsis was diagnosed in ICU	Survivors vs. death	b, c
Yunxia Chen	China	2014	2001SCCM/ESICM/ACCP/ATS/SIS	28-day mortality	prospective	680	502	178	within 1h after ED arrival	Survivors vs. death	a, b, c
Wei Zhang	China	2014	1992ACCP/SCCM	in-hospital mortality		58	34	24	day 1 and 3 after diagnosis	Survivors vs. death	a, b, c, d
Hwang Sung Yeon	Korea	2014	SIRS	28-day mortality	retrospective	591			within 3 h of ED admission		a, c
Young A Kim	Korea	2013	2005International pediatric SCC	28-day mortality	retrospective	65	48	17	admission to PICU	Survivors vs. death	a, b, c
Leonardo Lorente	Spain	2013	2001SCCM/ESICM/ACCP/ATS/SIS	30-day mortality	prospective	228	145	83	at the time of the diagnosis	Survivors vs. death	b, c
Nik Hisamuddin Nik Ab Rahman	Malaysia	2012	diagnosis	30-day mortality	prospective	41			admission to ED		a, c
Kana Ram Jat	India	2011	diagnosis	in-hospital mortality	prospective	30	15	15	admission to PICU	Survivors vs. death	a, b, c
P. Y. Boelle	France	2011	2001SCCM/ESICM/ACCP/ATS/SIS	14-day mortality	prospective	60			6h after ICU admission		c
Leonardo Lorente	Spain	2009	2001SCCM/ESICM/ACCP/ATS/SIS	in-hospital mortality	prospective	192	125	67	at the time of the diagnosis	Survivors vs. death	b, c
Alan E. Jones	USA	2009	diagnosis	in-hospital mortality	prospective	248	197	51	admission to ED	Survivors vs. death	a, b
C Vorwerk	England	2009	SIRS	28-day mortality	retrospective	307	235	72	admission to ED	Survivors vs. death	b, c
Mikkelsen	Canada	2009	2001SCCM/ESICM/ACCP/ATS/SIS	28-day mortality	retrospective	830	634	196	admission to ED	Non-shock vs. sepsis shock	c
Stephen Trzeciak	USA	2007	diagnosis	in-hospital mortality	prospective	1177					c
Charalambos A. Gogos	Greece	2003	1992ACCP/SCCM	in-hospital mortality	prospective	139	101	38	at admission	Survivors vs. death	b
T.D.Duke	Australia	1997	1990Septic shock in children	in-hospital mortality	prospective	31	21	10	at 0, 12, 24,48 h after admission	Survivors vs. death	b, c
G. Marecaux	Belgium	1996		in-hospital mortality		38	18	20		Survivors vs. death	b
J Bakker	Belgium	1991		in-hospital mortality		48	27	21		Survivors vs. death	b

SCC: Surviving Sepsis Campaign; MIMIC: Medical Information Mart for Intensive Care; SFAR-SRLF *: recommendations of the French Intensive Care Societies; APACHE: Acute Physiology and Chronic Health Evaluation; SCCM/ESICM/ACCP/ATS/SIS: Society of Critical Care Medicine/European Society of Critical Care Medicine/American College of Chest Physicians/American Thoracic Society, Surgical Infection Society. Assessment *: a: network analysis; b: mortality; c: different type of sepsis; d: diagnostic analysis. Superscript numbers are used to distinguish between two different studies by the same author.

**Table 2:** Risk of bias using the QUADAS-2.

Study	Risk of Bias				Applicability Concerns		
	Patient Selection	Index Test	Reference Standard	Flow and Timing	Patient Selection	Index Test	Reference Standard
Han Chen 2022	H	H	L	L	H	L	L
Ying Wu 2022	L	L	L	?	L	L	L
Yinjing Xie 2021	L	L	L	L	L	L	L
Junkun Liu 2021	L	L	L	L	L	L	L
Han Chen 2021	H	H	L	L	H	L	L
Jong Eun Park 2021	L	L	L	L	L	L	L
Gun Tak Lee 2021	L	L	L	L	L	L	L
Cristian Tedesco Tonial 2021	H	L	L	L	L	L	L
Yin Liu 2021	L	H	L	L	L	L	L
Ralphe Bou Chebl ^1^ 2021	L	H	L	L	L	L	L
Sarah M. Perman 2020	L	H	L	H	L	L	L
Amin Gharipour 2020	H	H	L	L	H	L	L
Yancun Liu 2020	L	L	L	L	L	L	L
Ralphe Bou Chebl 2020	L	H	L	L	L	L	L
Penzy Goyal 2020	H	L	L	L	H	L	L
Priyanka Jaiswal 2020	H	L	L	L	H	L	L
ShengYuan Hsiao 2020	L	L	L	L	L	L	L
Juhyun Song 2020	L	L	L	L	L	L	L
Nianfang Lu 2020	L	L	L	L	L	L	L
Haijiang Zhou 2020	H	H	L	L	H	L	L
Lifeng Wang 2020	L	L	L	L	L	L	L
Areesha Alam 2020	H	L	L	L	H	L	L
Narani Sivayoham 2019	L	H	L	L	L	L	L
Shengyuan Hsiao 2019	L	L	L	L	L	L	L
Yunlong Liu 2019	L	L	L	L	L	L	L
Chulananda D.A.Goonasekera 2019	H	H	L	L	H	L	L
Zhiqiang Liu 2019	L	H	L	L	L	H	L
Seung Mok Ryoo 2019	L	H	L	L	L	L	L
Ali Duman 2018	L	L	L	L	L	L	L
Zhengliang Peng 2018	L	L	L	L	L	L	L
Haipeng Yan 2018	H	H	L	L	H	H	L
Lama H Nazer 2018	H	L	L	H	H	L	L
Lefeng Zhang 2018	L	L	L	L	L	L	L
HsienHung Cheng 2018	L	H	L	L	L	L	L
Jikyoung Shin 2018	L	H	L	L	L	L	L
Ata Mahmoodpoor 2018	L	L	L	H	L	L	L
Chenggong Hu 2017	L	L	L	L	L	L	L
Julian Jimenez 2017	H	H	L	L	H	L	L
Helena Brodska 2017	L	L	L	L	L	L	L
Huaiwu He 2017	L	L	L	L	L	L	L
Richa Choudhary 2017	H	H	L	L	H	L	L
Walaa S. Khater 2016	H	L	L	L	H	L	L
Roberto Rabello Filho 2016	L	H	L	H	L	H	L
Esra Keçe 2016	H	L	L	H	H	H	L
Yanyan Zhou 2015	L	L	L	L	L	L	L
Wei Zhang 2014	L	L	H	L	L	L	H

L: low risk; H: high risk; ?: Unclear risk. Superscript numbers are used to distinguish between two different studies by the same author.

**Table 3:** Quality assessment of included studies by Newcastle–Ottawa Scale (NOS).

Study	Selection (Maximum 5 Stars)				Comparability (Maximum 2 Stars)	Outcome (Maximum 3 Stars)			Score (Maximum 10 Stars)
	Representativenes of Exposed Cohort	Selection of Non-Exposed Cohort	Exposure Ascertainment	Outcome Not Present at Start of Study	Comparability of Cohorts on the Basis of the Design or Analysis	Assessment of Outcome	Length of Follow-Up	Adequacy of Follow-Up	
Han Chen 2022	★	★	★			★	★	★	6
Lincui Zhong 2022	★	★	★		★★	★	★	★	8
Noa Galtung 2022	★	★	★	★	★★	★	★	★	9
Matteo Guarino 2022	★	★	★		★★	★	★		7
Yan Cao 2022	★	★	★	★	★★	★	★	★	9
Ying Wu 2022	★	★	★	★	★★	★	★	★	9
Yinjing Xie 2021	★	★	★		★★	★	★	★	8
Harith Alataby 2021	★	★	★		★★	★	★	★	8
Junkun Liu 2021	★	★	★	★	★★	★	★	★	9
Han Chen 2021	★	★	★			★	★	★	6
Jong Eun Park 2021	★	★	★	★	★★	★	★	★	9
Jongmin Lee 2021		★	★	★	★	★	★	★	7
Gun Tak Lee 2021	★	★	★		★★	★	★	★	8
Cristian Tedesco Tonial 2021		★	★		★	★	★	★	6
Xiaoyuan Wei 2021		★	★		★	★	★	★	6
Xiaonan Chen 2021	★	★	★			★	★	★	6
Qingbo Zeng 2021	★	★	★		★★	★	★	★	8
Hongsong Ma 2021	★	★	★		★★	★	★	★	8
Murat Erdogan 2021	★	★	★	★	★★	★	★	★	9
Utsav Nandi 2021	★	★	★		★★	★	★	★	8
Yin Liu 2021	★	★	★		★★	★	★	★	8
Ralphe Bou Chebl 2021	★	★	★	★	★★	★	★	★	9
Valentino D’Onofrio 2020	★	★	★	★	★★	★	★	★	9
Shuang Li 2020		★	★		★	★	★	★	6
S. Perez-San Martin 2020	★	★	★	★	★★	★	★	★	9
Amin Gharipour 2020		★	★			★	★	★	5
Yancun Liu 2020	★	★	★	★	★★	★	★	★	9
Oscar H. M. Lundberg 2020	★	★	★		★★	★	★	★	8
Ralphe Bou Chebl ^1^ 2020	★	★	★		★★	★	★	★	8
Ralphe Bou Chebl ^2^ 2020	★	★	★		★★	★	★	★	8
Romain Jouffroy 2020	★	★	★	★	★★	★	★	★	9
Penzy Goyal 2020		★	★		★	★	★	★	6
Haijiang Zhou 2020		★	★		★★	★	★	★	7
Tae Sik Hwang 2020	★	★	★		★★	★	★	★	8
Priyanka Jaiswal 2020		★	★	★	★	★	★	★	7
ShengYuan Hsiao 2020	★	★	★	★	★★	★	★	★	9
Juhyun Song 2020	★	★	★	★	★★	★	★	★	9
Keji Zhang 2020	★	★	★		★★	★	★	★	8
Nianfang Lu 2020	★	★	★	★	★★	★	★	★	9
Xiaomeng Tang 2020		★	★			★	★	★	5
Wen Li 2020	★	★	★	★	★★	★	★	★	9
Meryem Baysan 2020	★	★	★		★★	★	★	★	8
Filippo Mearelli 2020	★	★	★	★	★★	★	★	★	9
Haijiang Zhou 2020		★	★		★★	★	★	★	7
Lifeng Wang 2020	★	★	★		★★	★	★	★	8
Yusuke Hayashi 2020	★	★	★			★	★	★	6
Bernhard Wernly 2020	★	★	★			★	★	★	6
Areesha Alam 2020		★	★	★	★★	★	★	★	8
Gina Yu 2019	★	★	★		★★	★	★	★	8
Anitra C. Carr 2019	★	★	★		★★	★	★	★	8
Mudasir Nazir 2019		★	★	★	★★	★	★	★	8
Francesca Innocenti 2019	★	★	★	★	★★	★	★	★	9
Narani Sivayoham 2019	★	★	★	★	★★	★	★	★	9
Julian Villar 2019	★	★	★		★★	★	★	★	8
Ali Jendoubi 2019	★	★	★	★	★★	★	★	★	9
Jie Jiang 2019	★	★	★		★★	★	★	★	8
Shengyuan Hsiao 2019	★	★	★	★	★★	★	★	★	9
Elisa Estenssoro 2019	★	★	★	★	★★	★	★	★	9
Yunlong Liu 2019	★	★	★	★	★★	★	★	★	9
Anibal Basile-Filho 2019		★	★		★★	★	★	★	7
Han Li 2019		★	★		★★	★	★	★	7
Guillaume Dumas 2019	★	★	★	★	★★	★	★	★	9
Seung Mok Ryoo ^1^ 2019	★	★	★	★	★★	★	★	★	9
BoRa Chae 2019		★	★		★★	★	★	★	7
Steven J.Weiss 2019	★	★	★		★★	★	★	★	8
Sujay Samanta 2019	★	★	★	★	★★	★	★	★	9
Zhiqiang Liu 2019	★	★	★			★	★	★	6
Glenn Hernández 2019	★	★	★	★	★★	★	★	★	9
Seung Mok Ryoo ^2^ 2019	★	★	★		★★	★	★	★	8
Ali Duman 2018	★	★	★	★	★	★	★	★	8
Zhengliang Peng 2018	★	★	★		★★	★	★	★	8
Haipeng Yan 2018		★	★	★	★★	★	★	★	8
Lama H Nazer 2018		★	★		★★	★	★	★	7
Li Xing 2018	★	★	★	★	★★	★	★	★	9
Lefeng Zhang 2018	★	★	★		★★	★	★	★	8
HsienHung Cheng 2018	★	★	★		★	★	★	★	7
Jikyoung Shin 2018	★	★	★		★★	★	★	★	8
Takehiko Tarui 2018	★	★	★	★	★★	★	★	★	9
Ata Mahmoodpoor 2018	★	★	★	★	★★	★	★	★	9
Chenggong Hu 2017	★	★	★		★★	★	★	★	8
Julian Jimenez 2017		★	★	★	★★	★	★	★	8
Helena Brodska 2017	★	★	★		★★	★	★	★	8
Yongfeng Jia 2017		★	★		★★	★	★	★	7
Dong Hyun Oh 2017	★	★	★		★★	★	★	★	8
Motohiro Sekino 2017	★	★	★	★	★★	★	★	★	9
Aziz Kallikunnel Sayed 2017	★	★	★	★	★★	★	★	★	9
Adnan Javed 2017	★	★	★	★	★★	★	★	★	9
Mengshi Chen 2017		★	★		★★	★	★	★	7
Huaiwu He 2017	★	★	★		★★	★	★	★	8
Richa Choudhary 2017		★	★	★	★★	★	★	★	8
Juandi Zhou 2017		★	★	★	★	★	★	★	7
Luregn J. Schlapbach 2017		★	★		★★	★	★	★	7
KuanFu Chen 2017	★	★	★		★★	★	★	★	8
Kimie Oedorf 2016	★	★	★	★	★★	★	★	★	9
Roberto Rabello Filho 2016	★	★	★		★★	★	★	★	8
Ar-aishah Dadeh 2016	★	★	★	★	★★	★	★	★	9
Young Kun Lee 2016	★	★	★		★★	★	★	★	8
Aletta P. I. Houwink 2016	★	★	★		★★	★	★	★	8
Jan Philipp Bewersdorf 2016	★	★	★	★	★★	★	★	★	9
Sebastian A Haas 2016		★	★	★	★★	★	★	★	8
Sen Kuan Win 2016	★	★	★		★	★	★	★	7
Yanyan Zhou 2015	★	★	★		★★	★	★	★	8
Min Hyung Kim 2015	★	★	★		★★	★	★	★	8
Ivo Casagranda 2015	★	★	★	★	★★	★	★	★	9
Hao Wang 2015		★	★	★	★★	★	★	★	8
Leonardo Lorente 2014	★	★	★	★	★★	★	★	★	9
Yunxia Chen 2014	★	★	★	★	★★	★	★	★	9
Wei Zhang 2014	★	★	★		★★	★	★	★	8
Hwang Sung Yeon 2014	★	★	★		★★	★	★	★	8
Young A Kim 2013		★	★		★★	★	★	★	7
Leonardo Lorente 2013	★	★	★	★	★★	★	★	★	9
Nik Hisamuddin Nik Ab Rahman 2012	★	★	★	★	★★	★	★	★	9
Kana Ram Jat 2011		★	★	★	★★	★	★	★	8
P. Y. Boelle 2011	★	★	★	★	★★	★	★	★	9
Leonardo Lorente 2009	★	★	★	★	★★	★	★	★	9
Alan E. Jones 2009	★	★	★	★	★★	★	★	★	9
C Vorwerk 2009	★	★	★		★★	★	★	★	8
Mikkelsen 2009	★	★	★		★★	★	★	★	8
Stephen Trzeciak 2007	★	★	★	★	★		★	★	7
Charalambos A. Gogos 2003	★	★	★	★	★★	★	★	★	9
T.D.Duke 1997	★	★	★		★★	★	★	★	8
G. Marecaux 1996		★	★		★	★	★	★	6
J Bakker 1991		★	★		★	★	★	★	6

Each ★ indicates one score received in the according section. Superscript numbers are used to distinguish between two different studies by the same author.

## Data Availability

All data generated or analyzed during this study are included in this published article and its Supplementary Information Files. The datasets used and/or analyzed during the current study available from the corresponding author on reasonable request.

## Supplementary Materials

The following supporting information can be downloaded at: https://www.mdpi.com/article/10.3390/biomedicines12020447/s1, Figure S1. Sensitivity analysis of the individual trials on the results for blood lactate level associated with sepsis mortality; Figure S2. Sensitivity analysis of the individual trials on the results for blood lactate level associated survivors and non-survivors of sepsis; Figure S3. Funnel plot with Egger’s test for association between blood lactate levels and mortality; Figure S4. The network meta-analysis of available comparisons of blood lactate levels of patients with various outcomes; Figure S5. Comparison-adjusted funnel plot for blood lactate levels of patients with various clinical outcomes. A: nonsepsis; B: sepsis; C: severe sepsis; D: septic shock; Table S1. Original Sequential Organ Failure Assessment (SOFA) score; Table S2. Systemic Inflammatory Response Syndrome (SIRS) Criteria; Table S3. qSOFA (Quick SOFA) Criteria.

## Abbreviations

AUC	Areas under the curve
CI	Confidence interval
HSROC	Summary receiver operating characteristic curve
NOS	Newcastle–Ottawa Scale
OR	Odds Ratio
QUADAS	Quality assessment of diagnostic accuracy studies
SE	Standard error
SIRS	Systemic inflammatory response syndrome
SMD	Standard mean difference
SOFA	Sequential Organ Failure Assessment
SUCRA	Surface under the cumulative ranking
