# Supplementary material for: Hierarchical Capability in Distinguishing Severities of Sepsis via Serum Lactate: A Network Meta-Analysis
Source: Biomedicines. 2024 Feb 17;12(2):447. doi: 10.3390/biomedicines12020447 (PMC10886935; doi:10.3390/biomedicines12020447)
Supplement: Supplementary file 1 [file biomedicines-12-00447-s001.zip › biomedicines-2843609-supplementary.pdf]

Supplementary Materials

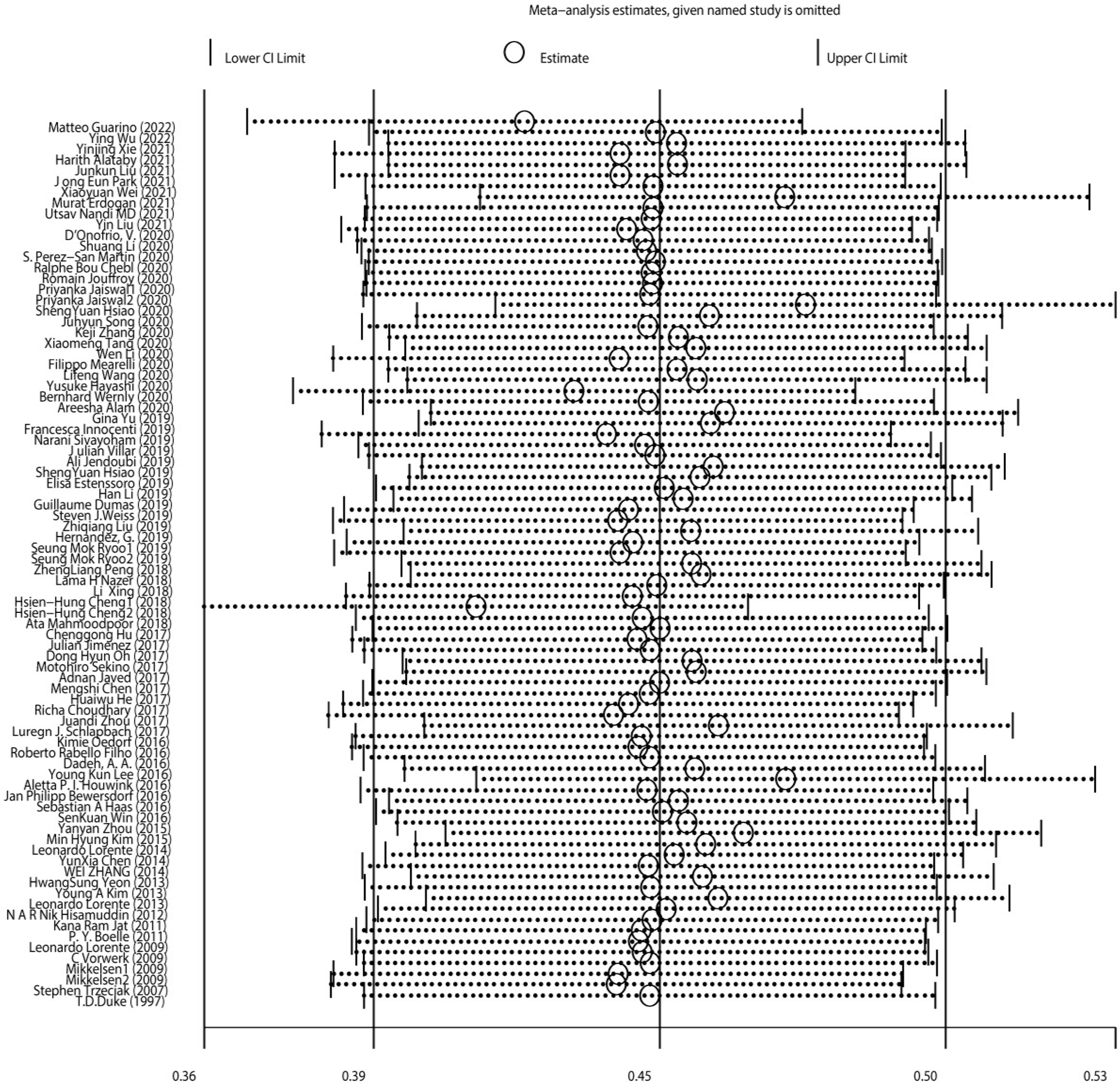

Supplementary Figure S1. Sensitivity analysis of the individual trials on the results for blood lactate level associated with sepsis mortality.

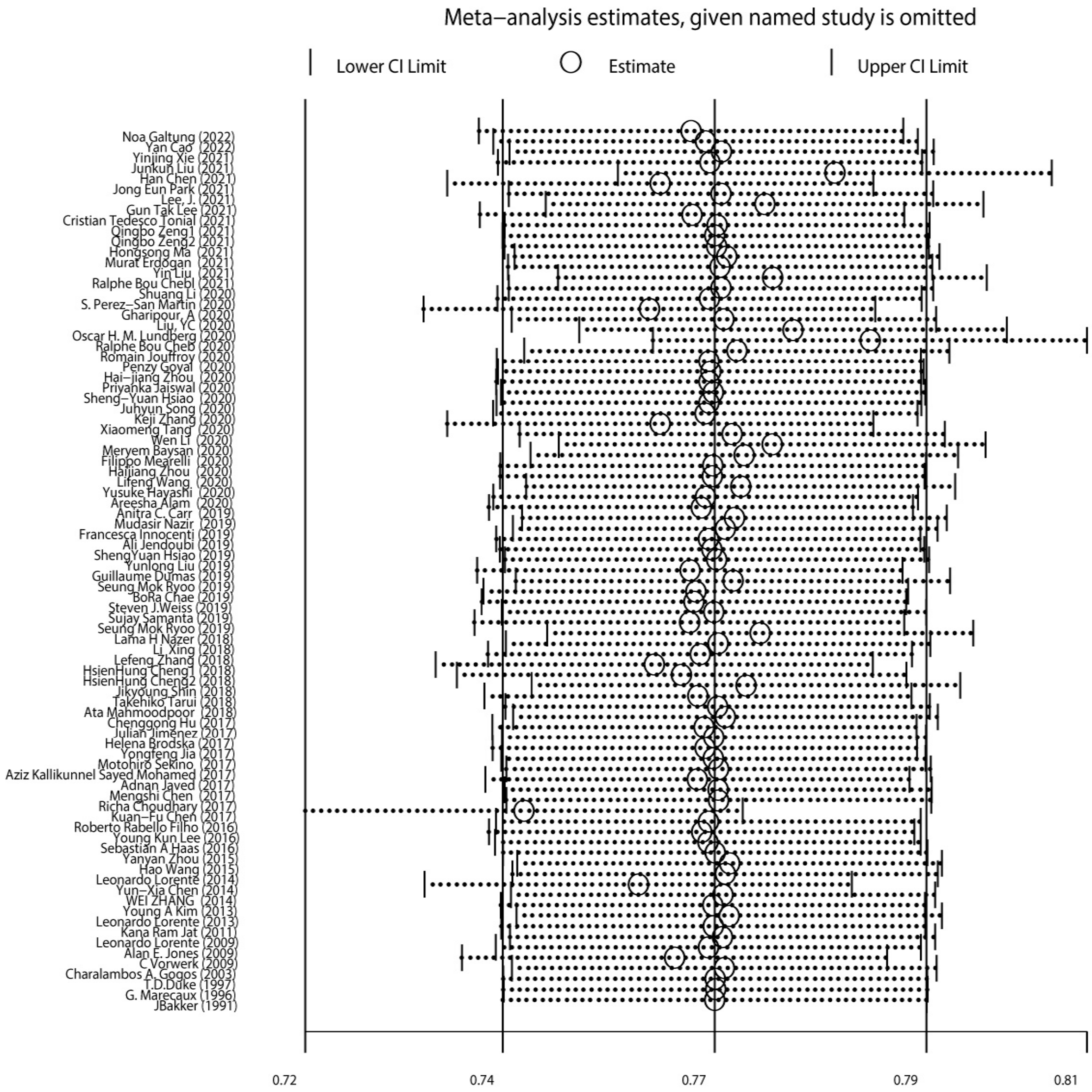

Supplementary Figure S2. Sensitivity analysis of the individual trials on the results for blood lactate level associated survivors and non-survivors of sepsis.

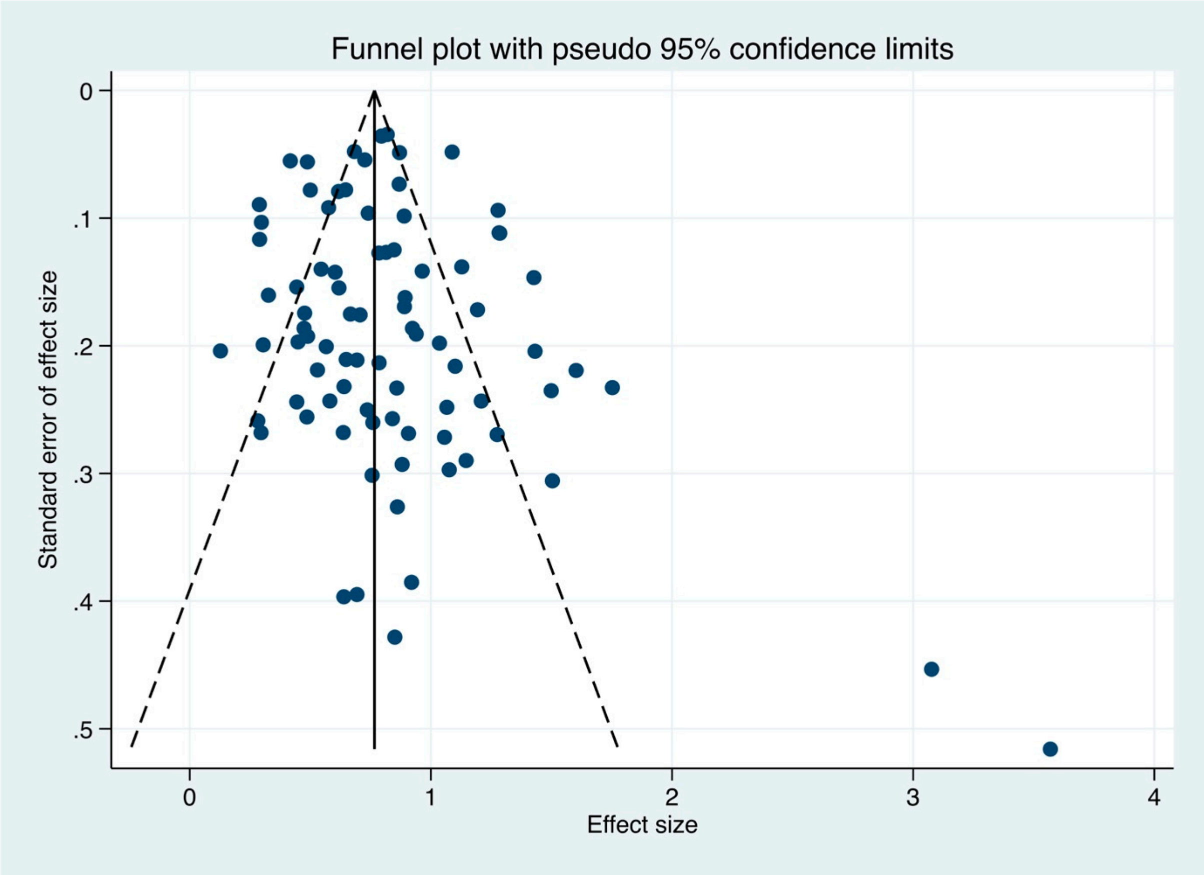

Supplementary Figure S3. Funnel plot with Egger's test for association between blood lactate levels and mortality.

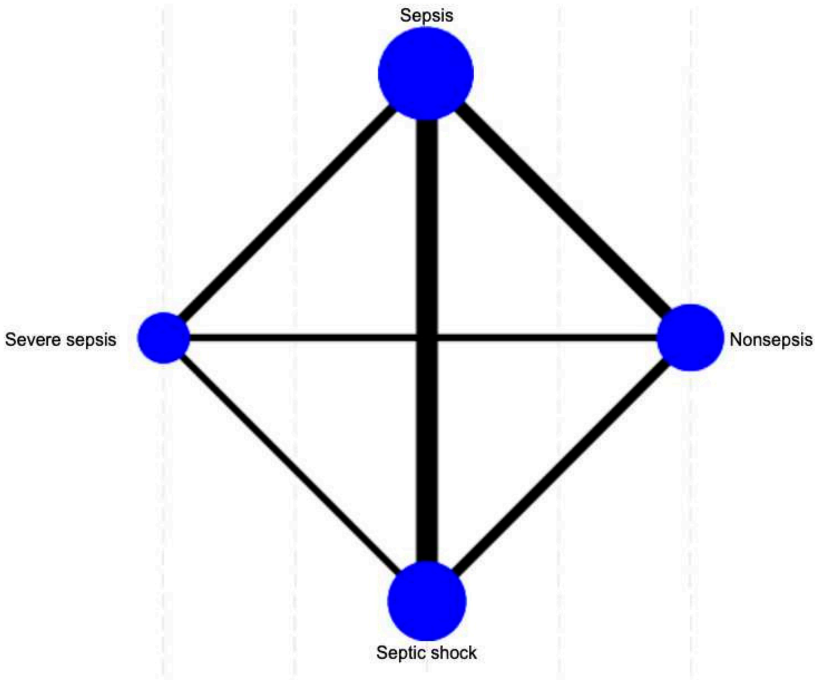

Supplementary Figure S4. The network meta-analysis of available comparisons of blood lactate levels of patients with various outcomes.

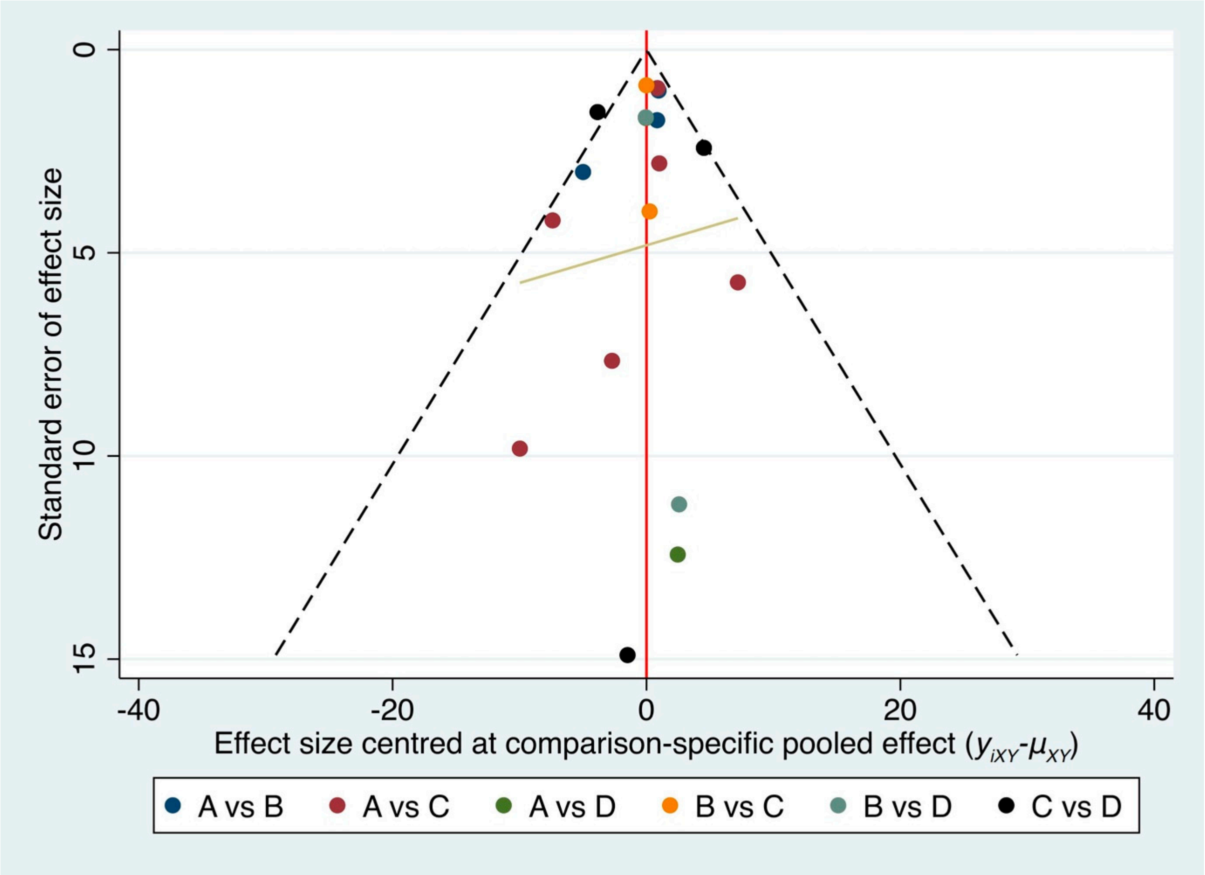

Supplementary Figure S5. Comparison-adjusted funnel plot for blood lactate levels of patients with various clinical outcomes. A: nonsepsis; B: sepsis; C: severe sepsis; D: septic shock.

Supplementary Table S1. Original Sequential Organ Failure Assessment (SOFA) score

System	Score 0	1	2	3	4
**Respiratory**					
$PaO2/FiO_2$,mmHg	≥400	<400	<300	<200 with respiratory support	< 100 with respiratory support
**Coagulation**					
Platelets × $10^3/mm^3$	≥ 150	100-149	50-99	20-49	< 20
**Liver**					
Bilirubin,mg/dL (μmol/L)	< 1.2 (< 20)	1.2–1.9 (20–32)	2.0–5.9 (33–101)	6.0–11.9 (102–204)	> 12.0 (> 204)
**Cardiovascular**					
Hypotension	No hypotension	MAP < 70 mmHg	Dopamine < 5 or dobutamine (any dose) *	Dopamine 5.1-15 or epinephrine ≤ 0.1 or norepinephrine ≤ 0.1*	Dopamine > 15 or epinephrine > 0.1 or norepinephrine > 0.1*
**Central nervous system**					
Glasgow Coma Scale score	15	13–14	10–12	6–9	< 6
**Renal**					
Creatinine, mg/dL (μmol/L) (or urine output)	< 1.2 (< 110)	1.2–1.9 (110–170)	2.0–3.4 (171–299)	3.5–4.9 (300-440) or UOP <500 mL/day	>5.0 (>440) or UOP <200 mL/day

FIO_2, fraction of inspired oxygen; MAP, mean arterial pressure; PaO_2, partial pressure of oxygen.

*Catecholamine doses are given as μg/kg/min for at least 1 hour.

Supplementary Table S2. Systemic Inflammatory Response Syndrome (SIRS) Criteria

Two or more of the following are required:
Body temperature >38°C or <36°C
Heart rate >90/minute
Respiratory rate >20/min or $PaCO_2$ <32 mmHg (4.3 kPa)
White blood cell count >12 000/mm^3 or <4000/mm3 or >10% immature bands

Sepsis = Infection + SIRS
Severe sepsis = Sepsis + evidence of organ dysfunction

Supplementary Table S3. qSOFA (Quick SOFA) Criteria

Two or more of the following are required:
Respiratory rate ≥22/min
Altered mentation (Glasgow Coma Scale score <15)
Systolic blood pressure ≤100 mm Hg
